# Special Supplement Introduction: Hallucinations

**DOI:** 10.1093/schbul/sbu043

**Published:** 2014-06-13

**Authors:** Charles Fernyhough, Flavie Waters

**Affiliations:** ^1^Hearing the Voice, c/o School of Education, Durham University, Durham, UK;; ^2^Department of Psychology, Durham University, Durham, UK;; ^3^Clinical Research Centre, Graylands Hospital, North Metro Health Service - Mental Health, Perth, Western Australia;; ^4^Centre for Clinical Research in Neuropsychiatry at the School of Psychiatry and Clinical Neurosciences, The University of Western Australia, Perth, Western Australia; ^5^ These authors contributed equally to this work.

**Keywords:** hallucinations, psychosis, interdisciplinary

## Abstract

This Special Supplement presents reports from 11 working groups of the interdisciplinary International Consortium on Hallucination Research meeting in Durham, UK, September 2013. Topics include psychological therapies for auditory hallucinations, culture and hallucinations, hallucinations in children and adolescents, visual hallucinations, interdisciplinary approaches to the phenomenology of auditory verbal hallucinations (AVHs), AVHs in persons without need for care, a multisite study of the PSYRATS instrument, subtypes of AVHs, the Hearing Voices Movement, Research Domain Criteria for hallucinations, and cortical specialization as a route to understanding hallucinations.

“What are voices?” At the World Hearing Voices Congress held in Melbourne, Australia in November 2013, this question was posed to a panel of experts by experience and experts by training. Each of the 9 panel members provided radically different answers, depending in large part on their personal experience with voices, clinical and research backgrounds, and philosophical and cultural perspectives. The debate sharply illustrated the disparate views held about hallucinations among the research and clinical communities, and in the general population.

For an understanding of a complex and heterogeneous phenomenon such as hallucinations, it is essential to combine the knowledge drawn from different perspectives and disciplines. The International Consortium on Hallucination Research (ICHR), which held its inaugural meeting in London in 2011, represents a new approach to the study of hallucinations which posits that close collaborations between experts with different pockets of knowledge can considerably enhance our understanding of this fascinating and multifaceted aspect of human experience.

The articles in this special supplement of *Schizophrenia Bulletin* comprise the reports of 11 working groups who worked on different domains of investigation relating to hallucinations, each summarizing the available knowledge, gaps in their respective fields, and ideas for future research directions. Their findings were presented at the ICHR meeting on September 12–13, 2013, at Durham University, UK.

In preparing for this second ICHR meeting, we were keen to extend the range of interests covered by its inaugural meeting. Partly as a result of our being hosted by the Hearing the Voice project, the outcome was a more holistic approach to hallucinatory phenomena, incorporating social and cultural as well as personal, cognitive, and neuroscientific perspectives. Hearing the Voice is an interdisciplinary project (funded by the Wellcome Trust) examining the phenomenon of auditory verbal hallucinations (AVHs) from a range of different disciplinary perspectives. The project adopts a medical humanities approach aimed at integrating first-person and third-person perspectives on hallucinations, through an ambitious coarticulation of scientific and humanities traditions.

In preparation for the Durham meeting, we were also guided by the recommendations from the first Consortium meeting.^1^ In addition, we were mindful of trying to cover important perspectives such as that of the Hearing Voices Movement, as well as new initiatives that had emerged since the first ICHR meeting, including the development of National Institute of Mental Health (NIMH) Research Domain Criteria (RDoC) for hallucinations. We coordinated the establishment of 12 working groups on various topics related to hallucinations. Eleven groups report here; one will report at another date.

In the first article, Thomas et al^2^ consider the current status and future directions in research on psychological therapies targeting auditory hallucinations (voices). They discuss the development of, and empirical evidence for, therapies for voices, and examine possible new research directions. Next, Larøi et al^3^ discuss the role of culture in shaping hallucinations. Drawing on the expertise of anthropologists and psychologists, they explore ways in which culture impacts on the phenomenology, understanding, and labeling of these experiences. In the third article, Jardri et al^4^ bring together knowledge on hallucinations in children and adolescents. They provide a review of epidemiological and risk factors, and cognitive and brain imaging studies, as well as key issues that have interfered with progress.

Waters et al^5^ then provide a review of visual hallucinations in the psychosis spectrum, examining prevalence, phenomenology, clinical characteristics, and assessment methods alongside studies of cognition, brain imaging, electrophysiology, and treatment. These findings are compared with the literature drawn from neurodegenerative disorders and eye disease. In the fifth article, Woods et al^6^ consider the value of adopting an interdisciplinary approach to the phenomenology of AVHs, including benefits for understanding the heterogeneity of the experience, informing studies in cognitive neuroscience, and suggesting new possibilities for therapeutic intervention.

Johns et al^7^ then provide a synthesis of the literature on AVHs that occur in the context of the general population with no identifiable disorder. They also discuss the similarities and key differences in phenomenological features and underlying mechanisms in individuals with and without the need for care. In the seventh article, Woodward et al^8^ present an investigation of the underlying constructs of the PSYRATS in individuals with psychosis, combining data from 12 sites in 7 countries, and proposing a new 4-dimensional model for auditory hallucinations.

McCarthy-Jones et al^9^ then consider the evidence for distinct subtypes of AVHs, proposing 5 main categories of the experience that have implications for research design and clinical interventions. Corstens et al^10^ discuss the key values of the international Hearing Voices Movement, its historical growth, and implications of its values for research and practice. Ford et al^11^ investigate how the recent NIMH RDoC framework can be used to understand the experience of hallucinations. They consider transdiagnostic and cross-population comparisons and ask how different RDoC domains might be relevant to the study of hallucination phenomenology. In the final paper, ffytche and Wible^12^ review the evidence linking cell properties and content as a starting point for a theory of how cortical specialization may help understanding different modalities of simple and complex hallucinations.

Now in its fourth year, the ICHR is increasing in size and gathering speed owing to the growing interest of leading researchers and experts by experience. The next meeting of the ICHR is to be held in Melbourne in October 2015, requiring the formation of new working groups. A satellite “open” meeting of the ICHR is being held in Norway in September 2014, featuring presentations from the research and clinical community and experts by experience. Further information about the latest meeting information is available from hallucinationconsortium.org.

We are grateful to the Wellcome Trust for their financial support of the Durham ICHR meeting and the publication of this Special Supplement.

## Funding

Wellcome Trust grant (WT098455 to C.F.).
